# Nudge theory and gambling: a scoping review

**DOI:** 10.3389/fpubh.2024.1377183

**Published:** 2024-06-10

**Authors:** Marie-Ève Fortier, Sophie Audette-Chapdelaine, Anne-Marie Auger, Magaly Brodeur

**Affiliations:** ^1^Department of Psychology, University of Sherbrooke, Sherbrooke, QC, Canada; ^2^Department of Family and Emergency Medicine, University of Sherbrooke, Sherbrooke, QC, Canada

**Keywords:** gambling, nudge, dark nudge, behavioral economics, public health, scoping review

## Abstract

**Background:**

Gambling disorder (GD) is a pressing public health concern with significant societal costs. The recently developed nudge theory, which is rooted in behavioral economics, aims to influence the decision-making behaviors of individuals by implementing changes in the environment.

**Aim:**

This scoping review aims to synthesize the literature on nudge theory as it relates to gambling.

**Methods:**

This scoping review accords with the Arksey and O’Malley framework, as refined by Levac et al. It includes only articles from peer-reviewed journals that focus, as main themes, on both nudge theory and gambling. The final study selection includes six articles.

**Results:**

The scoping review process led to studies explaining how (1) nudges aim to prod people toward healthier gambling choices, fostering the adoption of more responsible gambling practices, and (2) some gambling features, called dark nudges (or sludges), exploit and harm the decision-making processes of people who gamble.

**Conclusion:**

This scoping review highlights the fact that many stakeholders are involved in the field of gambling, and that better cooperation between them would promote safer and more responsible gambling practices. Future research is also needed to empirically test nudges to develop a better understanding of their impact on those who gamble.

## 1 Introduction

In the Diagnostic and Statistical Manual of Mental Disorders, Fifth Edition (DSM-5) (1), gambling disorder (GD) is classified in the “Substance-Related and Addictive Disorders” category and is defined as “persistent and recurrent problematic gambling behavior leading to clinically significant impairment or distress” (1). Previous studies have concluded that other mental health disorders are often comorbid with GD, such as substance use disorders, depressive disorders, anxiety disorders, and personality disorders (1–3).

GD is a major public health issue (4), giving rise to many harms that often impact both those who gamble and the people close to them, such as family and friends (5). The classification of gambling harms by Langham et al. (5) illustrates that GD can lead to numerous problems: financial harms, relationship disruptions, emotional or psychological distress, decrements in health, cultural harms, reduced performance at work or school, and increased criminal activity.

Over the last few decades, many measures have been implemented to reduce the harms associated with GD. In many sectors, such as the healthcare (6, 7) and substance abuse (8) systems, the efficacy of nudges has been evidenced. The concept of nudge was introduced in 2008 by Thaler and Sunstein (9), and has been useful in promoting public health while preserving individual freedom and free choice. The authors described nudges as “any aspect of the choice architecture that alters people’s behavior in a predictable way without forbidding any options or significantly changing their economic incentives” (9). More precisely, according to choice architecture the decision-making process is influenced by the way that the choices are displayed (10). This school of thought falls under behavioral economics, which is a field at the intersection of economics and psychology that aims to understand and explain human decision-making processes (11). While nudges are supposed to influence positively the decision-making process, another side of nudge theory, called dark nudges or sludges, tends to be prejudicial (12). Moreover, these kinds of nudges are frequently applied in the gambling field (12).

Sylvester and Booch (8) conducted a realist review of the efficacy of nudge theory in reducing alcohol and tobacco consumption. Despite a limited number of studies, the review identified four effective interventions, indicating a positive impact, although these offer no guarantees (8). However, the application of nudges in the gambling sector remains unexplored. What is the current understanding of the potential effectiveness of nudges in this context?

Given the significance of GD as a critical public health concern and the promising potential of nudge theory in addressing addictive behaviors, the objective of this scoping review was to comprehensively examine the existing peer-reviewed scientific literature that focuses on the intersection of nudge theory and gambling. The following research question guided this scoping review: “What is the current state of scientific knowledge on the relationship between nudge theory and gambling?”

## 2 Methods

This scoping review is based on the methodological framework of Arksey and O’Malley (13) as refined by Levac et al. (14). It includes the following five steps: (1) identifying the research question, (2) identifying relevant studies, (3) selecting the studies, (4) charting the data, and (5) collating, summarizing, and reporting results (14).

### 2.1 Data sources and search strategy

**Figure 1:**
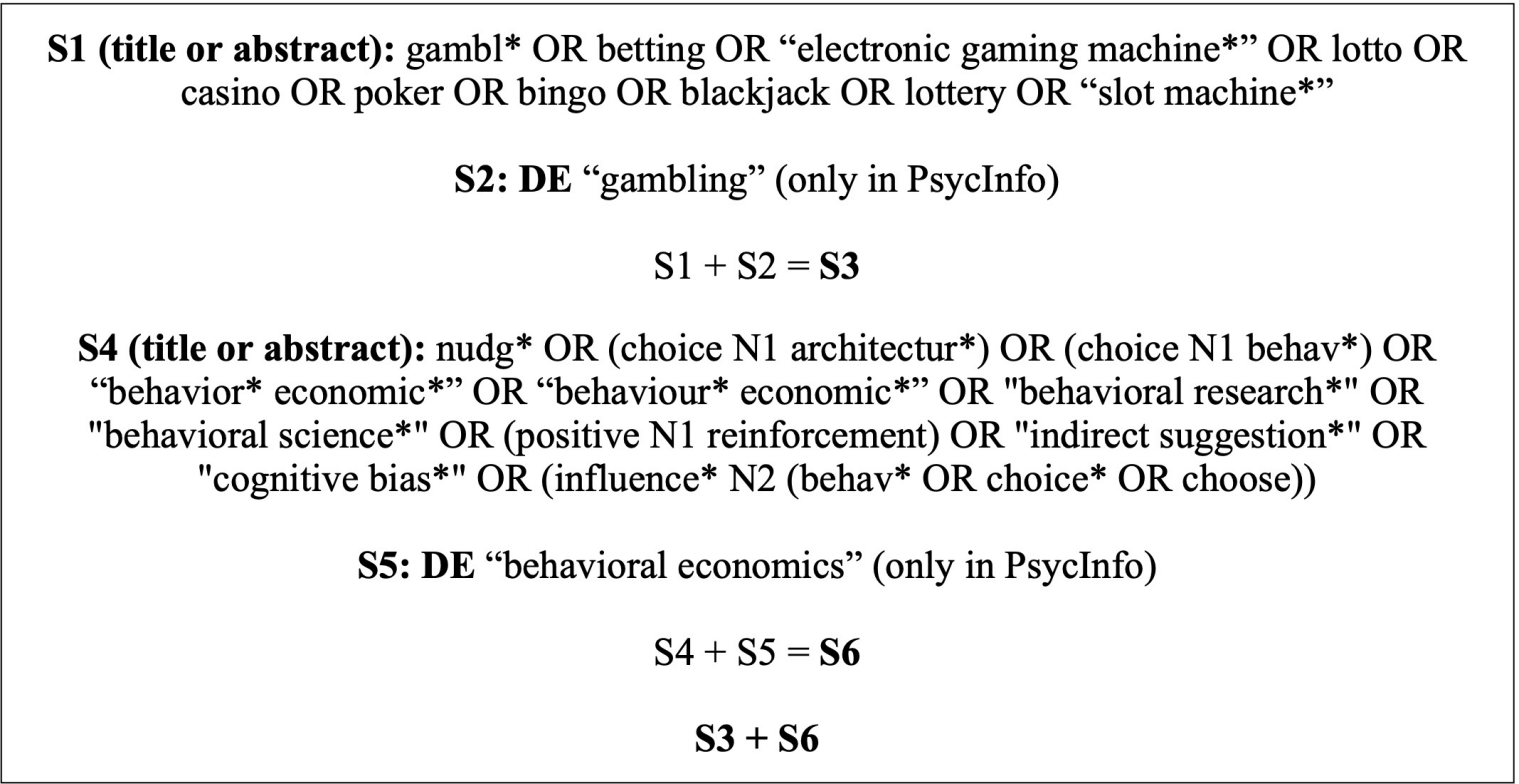
Search strategy employed to perform a literature search across nine electronic databases.

A literature search was performed on August 3, 2023, in nine electronic databases via the EBSCOhost platform: APA PsycINFO, Academic Search Complete, APA PsycExtra, Business Source Complete, CINAHL Plus with Full Text, EconLit with Full Text, MEDLINE with Full Text, Psychology and Behavioral Sciences Research Topic, and SocINDEX with Full Text. The search strategy included terminology from the concepts of both “nudge” and “gambling” (see Figure 1) and was developed with the help and support of an academic health librarian from the University of Sherbrooke. In addition, the terms “nudge” and “gambling” were searched on Google Scholar and the references of all selected articles were closely examined.

### 2.2 Study selection

The following inclusion criteria were applied during article selection for this scoping review: (1) articles are directly about the concept of nudge and gambling, (2) articles are published in a peer-reviewed journal, and (3) articles are in English.

**Figure 2:**
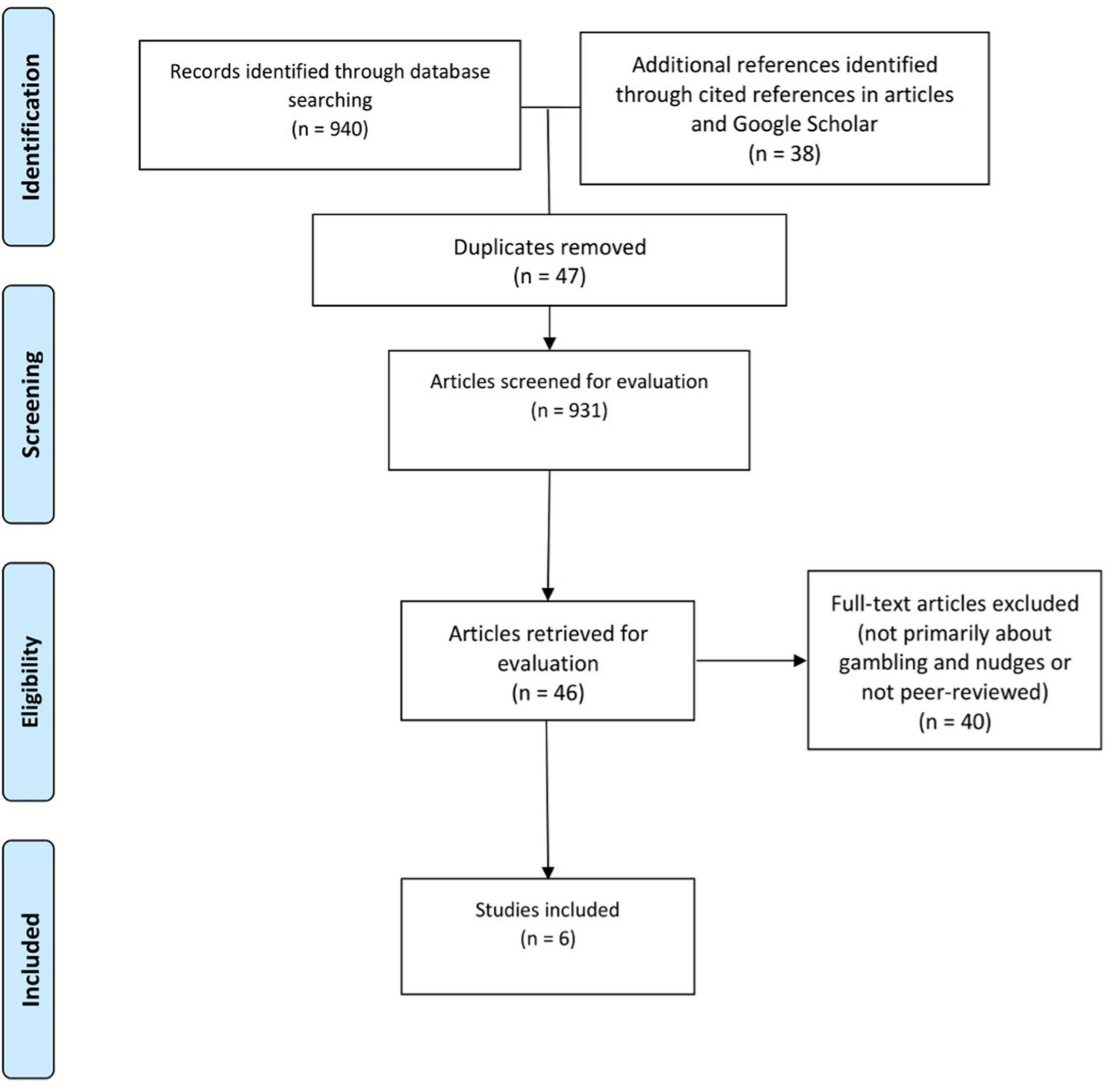
PRISMA flow diagram summarizing identification, screening and evaluation of articles resulting from the initial literature search.

The sorting of the resulting 978 articles began with the withdrawal of duplicates, leading M-EF to screen the titles and abstracts of 931 articles. M-EF then reviewed the remaining 46 full-text articles and classified each according to our level of certainty about its relevance for our scoping review and its accordance with the inclusion criteria. During the selection process, disagreements were discussed by all team members. M-EF, SA-C, and MB debated the inclusion or exclusion of the last four articles under evaluation. This concluded the final selection of the six articles included in our study. All team members approved the final selection (Figure 2).

### 2.3 Data extraction and data analysis

M-EF conducted the data extraction of the following characteristics for the six articles: authors’ names, year, title, country, objective, methodology, population studied, and main conclusions of the article (see Table 1). Table 1 was revised by SA-C and MB. The data collected in the six articles were grouped by themes and are presented according to the narrative synthesis approach, which consists of concise “findings from multiple studies that rely primarily on the use of words and text to summarize and explain the findings” (21).

**Table 1:** Description of the studies included in the scoping review.

Authors, Year	Title	Country	Type	Objective	Methodology	**Population and age**	**Conclusions**
Gainsbury et al. (11)	Behavioral Economics and Gambling: A New Paradigm for Approaching Harm- Minimization	Australia	Theoretical article	“The current article proposes that the field of behavioral economics offers a valuable perspective for the gambling and broader addiction fields. This article focuses on understanding the heuristics and biases that drive behavior with an aim of guiding the development of effective interventions to minimize gambling-related harms.”	N/A	N/A	“Through understanding how psychology influences decision making, a behavioral economics approach offers promising interventions to minimize gambling-related harms across the spectrum of gambling risk. The appeal of nudging is self-evident: it proposes a set of seemingly subtle, low-cost environmental and policy changes that can be applied to a wide range of individuals, or targeted groups. However, in gambling, a greater evidence base is required, including both primary research and the evaluation of existing evidence where it is available. [...) Greater awareness of the impact of nudges and behavioral economic interventions by gambling regulators and policymakers may lead to greater and safer regulation of game design, and enhanced gambling harm prevention and treatment initiatives.”
Murch and Clark (17)	Commentary on Graydon et al. (16): Realistic simulations and nudging gambling policy	Canada	Commentary	Explore the contribution of the Graydon et al. study about losses disguised as wins in multi-line slot machines.	N/A	N/A	“Highly realistic slot machine simulations are a powerful way to test the impact of gambling structural features. ‘Nudging’ may provide a framework to mitigate the effects of Losses Disguised as Wins and, more broadly, to translate research findings into gambling policy. [...) emerging lessons from the field of behavioral insights may provide innovative new ideas, and ways to enhance existing tools, in order to mitigate gambling harms.”
Newall (18)	Dark nudges in gambling	United Kingdom	Editorial	“In this editorial I argue that, in gambling, nudging works differently. Gambling’s ‘dark nudges’ are designed to exploit gamblers’ biases, as economic rationality on the part of gambling firms predicts. Gambling’s dark nudges reveal the contradictions of industry-led responsible gambling initiatives, and show how stronger regulation is required to reverse gambling’s spiraling public health costs.”	N/A	N/A	“While the gambling industry claims to support responsible gambling, the action of these same firms’ dark nudges speak louder than words. And responsible gambling messages only increase gamblers’ perceived stigma; a cruel irony given how the system is designed to exploit them. [...) Gamblers are not helped by some governmental actors who hesitate over gambling restrictions because of short-run revenue losses, despite the large costs of gambling to society.”
Gainsbury et al. (15)	Reducing Internet Gambling Harms Using Behavioral Science: A Stakeholder Framework	Australia	Perspective	“This paper presents a framework for how behavioral science principles can inform appropriate stakeholder actions to minimize Internet gambling-related harms.”	N/A	N/A	“Collaborative efforts between stakeholders could result in the implementation of appropriate design strategies to assist individuals to make decisions and engage in healthy, sustainable behaviors.”
Newall and Rockloff (19)	Promoting safer gambling via the removal of harmful sludge: a view on how behavioral science’s “nudge” concept relates to online gambling	Australia	Letter to the editor	“Here, we argue that online gambling operators’ actions are more consistent with sludge than nudge, and that sludge reduction shows more current promise for promoting safer gambling”	N/A	N/A	“Although it would be beneficial to nudge gamblers toward safer choices, the prevention of both current and potential sludge practices should be of higher urgency in the agendas of those who want to promote safer gambling”
Pennay et al. (20)	Sports bars: environmental design, drinking, and sports betting	Australia	Original Research	“[...) the purpose of this article is to investigate the physical design of, and practices enacted within, sports bars to provide some indication as to whether these venues are spaces that encourage risky drinking and betting.”	Mixed-method “Our analysis draws on three arms of data Research Topic: (i) website review of licensed venues in Victoria, Australia that promoted sports telecasting; (ii) observational data Research Topic including site visits to 50 venues in Victoria that promoted sport telecasting, and more intensive sessions of observation during six sporting matches; and (iii) *in situ* interviews with 200 sports bar patrons.”	Venues in Victoria, Australia (*n* = 50), Sports bars (*n* = 9), Has a sports bar section (*n* = 22) Just televises sports (*n* = 19) Patrons in venues (*n* = 200) 70% male, 30% female, Age mean = 33.3	“We identified several design elements across the three types of venues, each ‘nudging’ sports betting and drinking in different ways. Any focused public health work in relation to sports bars will need to consider the social and physical design elements of different sports betting environments.”

## 3 Results

### 3.1 Study characteristics

All selected articles were published between 2018 and 2021, and four of the six were from Australia (11, 15, 19, 20). The other two articles were from Canada (17) and the United Kingdom (18).

### 3.2 Article type

There was wide variety within our selection of articles, comprising a theoretical article (11), a commentary (17), an editorial (18), a perspective (15), a letter to the editor (19), and one original research study (20) (see Table 1).

Regarding the methodology and the characteristics of the populations studied, only the original research study provided such information (20). As this is mixed-method research, it adopts a mixed quantitative and qualitative design. The data analyzed came mostly from observations performed in sports bars, as well as interviews with customers. The mean age of the studied group was 33.3 years old, and the sample was composed principally of men (70%).

### 3.3 Behavioral economics

The article by Gainsbury et al. (11) explains behavioral economics in the most detail. Indeed, the authors mention that, according to behavioral economics, individuals tend to use shortcuts known as heuristics in complex and uncertain situations. Heuristics enable individuals to make prompt decisions with less mental effort. However, this can also generate unconscious errors, called biases. The authors also report that many of these heuristics and biases are exploited in gambling. Two articles in our selection (11, 18) explicitly link gambling features to heuristics and/or biases, giving examples as to how gambling games are conceptualized with strategies such as “losses disguised as wins” or “near-misses.” These strategies can take the form, for example, of a slot machine displaying a congratulatory message for a win that is worth less than the bet (11). Murch and Clark (17) also note that “losses disguised as wins” harm the decision-making process for people who practice gambling. Two articles in our study selection also briefly mention the field of behavioral science (15, 19), while one makes no mention of it (20).

### 3.4 Nudges

All six selected articles define the nudge concept as deriving from choice architecture (see Table 1). As mentioned by Gainsbury et al. (11), nudges “involve making subtle alterations in the choice environment to encourage behavior change.” Unlike dark nudges, nudges aim to prompt people into making healthier choices (17, 19, 20). Nudges are also characterized by not restricting people, who remain free to choose other options (15, 17).

In the gambling context, the authors mention that nudges could be used to lead individuals to adopt healthier (15) and safer (19) gambling practices. The effectiveness of nudges in gambling lies in their cost-effectiveness and subtlety (11). Nudges in gambling are most effective when they resonate with individuals’ own understanding of what is important (11).

As seen in Table 2, some examples of nudges in gambling involve the use of normative feedback to compare one’s gambling behaviors with that of others. Such feedback could also include incentives (such as smiley faces, words of encouragement, etc.), dynamic messages during gambling (“Do you need to take a break?”) (11), or a monthly summary of all gambling activities (11). Murch and Clark (17) suggest that it would be beneficial to use nudge theory to modify slot machines, such as by suppressing the sound normally made for “losses disguised as wins.”

**Table 2:** Examples of nudges and dark nudges in the literature.

Authors, year	Definition of nudges	Examples of nudges in gambling	Definition of dark nudges/suldges	Examples of dark nudges/sludges in gambling
Gainsbury et al. (11)	It “involve[s) making subtle alterations in the choice environment to encourage behavior change.”	- Normative messages (i.e., “[...) activity statements with a clear summary of their monthly net gambling outcome that include a statement or graphic comparing their gambling outcomes with the average bettor”). - “Nonfinancial penalities” - “[...) Incentives like smiley faces and words of encouragement.” - “[...) personalized messages that target players based on individual characteristics and patterns of play.” - Dynamic messages (i.e., “Do you need to take a break?”). - “As a default option, credit card companies could block expenditure at gambling venues and sites, and require consumers to opt out of the block by notifying their financial institution if they wish to enable gambling expenditure at certain venues or sites.” - “Gambling operators can set conservative deposit or money transfer limits, lower the default bet size option of a game, or send players’ winnings to a separate “cash out” account, with players having to select to re-gamble their money.” - “[...) cues encouraging people to think about the benefits of saving funds.” -” [...) pre-setting limits on the time and/or amount of money spent (lost) gambling. [...) Planning prompts and reminders to encourage people to follow through with their preferred and pre-stated course of action can also help facilitate and enhance adherence to desired behavior.”	N/A	- “[...) stopper buttons on slot machines encourage illusory control beliefs.” - “Roulette games display a history of red/black outcomes, which fosters the gambler’s fallacy [...)” - “slot machines provide losses disguised as wins whereby the player is congratulated for a win, with the value being less than that of the bet” - “[...)"Bet again” button [...) saves a player’s previous bet size and play-line(s) choice [...).” -"The transfer of cash into credit or chips also facilitates flow, by way of it being easier to keep gambling than return to the cashier to exchange chips or credits for money.”
Gainsbury et al. (15)	“Nudge theory uses choice architecture and choice framing to ask questions in a way that nudges individuals’ behavior in certain directions without restricting the available options [...).”	- “[...) personalized [and) normative feedback [...).” - “[...) encouragement to moderate play through pre-commitment devices.” -"Dynamic messages can create a break in play and encourage self-appraisal.” -”[...) alarm clocks and ring-fenced winnings to prevent re-gambling.” -"Digit wallets can limit gambling expenditure and provide personal feedback on gambling spend.” - “Design options may include “plain packaging” for gambling sites (minimizing color and graphics), increasing friction by requiring users to click through different pages to access different betting/game options, creating pauses to slow the betting speed, reducing defaults bets, and requiring users to confirm bets and manually entering the amount, using default automated withdrawals of winnings, and default opt out of notifications and marketing.”	N/A	- “[...) push notifications of time-limited promotional offers [...).” - “[...)matched deposits with complicated terms and conditions and limited benefits for users [...).” - “[...) excessive friction creating difficulty in withdrawing deposited funds [...).” - “[...) targeted push messages promoting a betting or spending options matching the user’s profile (“people like you bet on...”) [...)” - “[...) encouraging countinuous use by eliminating natural breaks in play or the ability to pause (e.g., infinity scrolling).” - “[...) dynamic leader boards of recent winners [and) money back guarantee bets [...).” - “[...) providing details of previous wins in independant events such as winning lottery or roulette numbers, time since last jackpot, location winning lottery tickets were sold [...).” - [...) promoting irrelevant information [...) [such as) most popular bets, number of active users [...).” - “[...) prompted bet size, frictionless betting.”
Murch and Clark (17)	“The zeitgeist in public health and policy-oriented research emphasizes ‘nudging’ consumers to make better, healthier choices. Nudges influence behavior without limiting the ability to choose alternative options or significantly altering economic incentives.”	“Receiving timely, task-relevant information [...).”	“‘Dark nudge’ or ‘sludge’ [are) an influence that obstructs (rather than aids) good decision-making.”	- “Losses disguised as wins” (LDWs)
Newall (18)	“‘Nudge’ has come into common usage in behavioral science, the intersection of psychology and economics, for situations where a ‘choice architect’ aligns a system with consumers’ best long-term interests.”	- “Warning messages have often been added to dangerous gambling products [...).” - “Responsible gambling messages [...).”	“Gambling’s ‘dark nudges’ are designed to exploit gamblers’ biases, as economic rationality on the part of gambling firms predicts.”	- “Large denominations of money, or token equivalents, are inserted for a continuous gambling experience.” -"Touchscreen buttons minimize the physical effort of long gambling sessions.” - “Near-miss” - “Losses Disguised as Wins” -”[...) meaningless bells, whistles, and associations [...).”
Newall and Rockloff (19)	“‘Nudge’ is a key concept of behavioral science, which is being used by governments to encourage better decision making. [...) Nudges improve consumer welfare via encouragements rather than mandates, and have been suggested as ways of promoting safer gambling by academics [...).”	N/A	“‘Sludge’ [are) attempts to profit by encouraging consumers to act against their own best interests; a term synonymous with what has previously been called a ‘dark nudge’.”	- “Deposits could be made instantaneously, but withdrawals would only be processed after several days, during which time they could be subject to a customer-initiated cancellation: a ‘reverse withdrawal’. This asymmetry in favor of deposits over withdrawals is consistent with sludge [...).” - “High suggested deposit limits” - “[...) how mandated cost-of-play is placed in a frequently misunderstood format at the bottom of difficult to navigate help screens.”
Pennay et al. (20)	“One of the main goals in the choice architecture literature is to explore ways in which it can be used to nudge people to make ‘better’ or ‘healthier’ choices [...).”	N/A	“[They are) designs that encourage consumers to make choices that may not be ‘healthier’ or ‘better’ for the individual, but instead serve the commercial interests employing the choice architect. [...) [They are) features that are intended to exploit gamblers’ preferences or biases to maximize economic gain.”	- “[...) features such as crowding, layout, lighting, color shemes, music and ambience.” - “[...) given the prominence of [electronic gaming machines) in these [venues that have a sports bars)– noted by observers in terms of visibility, lighting, scale and proximity to the entrance – it is possible that sports betting patrons may be nudged (perhaps even deliberately) into [electronic gaming machine) use.”

Newall and Rockloff identify a nuance about nudges in gambling (19). While they agree that nudges could prompt safely gambling practices, they consider that it would be more urgent to reduce the dark nudges present in gambling environments.

Finally, while all of the articles discussed nudges, two of them defined the concept of nudges only briefly, after which they focused on dark nudges (18, 20).

### 3.5 Dark nudges and sludges in gambling

In four of the six selected articles, the authors explicitly question the dark nudges features of gambling games, as these impair the decision-making processes of those who gamble, thereby taking advantage of their biases (see Table 2) (17–20).

Murch and Clark (17) consider “losses disguised as wins” to be dark nudges. Newall and Rockloff (19) also give examples of these features in gambling, such as suggesting high deposit limits. Pennay et al. (20) observed elements in sports bars that could also be considered dark nudges, such as the “visibility, lighting, scale and proximity to the entrance” of the electronic gaming machines. In addition, Gainsbury et al. (15) mention that some features in gambling, such as “infinity scrolling,” nudge users into “continued gambling.” These words dovetail with the concept of dark nudges, even though the term itself is not used by the authors.

### 3.6 Stakeholders

Many stakeholders are involved in gambling, and some of the articles in our selection discuss their roles (15, 18, 19), with some reporting on the contradictions or conflicts of interest that exist among them (11, 15). Two articles—Gainsbury et al. (11, 15) and Newall (18)—report that stakeholders should work together to reduce gambling harms. More precisely, Gainsbury et al. (15) provide an in-depth description of the roles played by individual users, community groups, gambling industry operators, government actors and regulators, financial institutions, and researchers to establish safer online gambling. Newall (18) discusses briefly the dynamics between stakeholders and notes that “gamblers, gambling firms, regulators and researchers” are all implicated in the current state of the gambling environment.

Gainsbury et al. (11) and Newall (18) note that while gambling is a source of government income, it comes with a big societal cost, and this cost causes a conflict of interest (11). Newall (18) adds that the presence of dark nudges in gambling is a mark of contradiction from gambling officials, since “the gambling industry claims to support responsible gambling” (18).

### 3.7 Future of nudges in gambling

All of the articles in our selection advise that actions be taken to encourage responsible gambling or to reduce gambling harms. In accord with the fact that their articles mostly address dark nudges, the recommendations of Newall (18) and Pennay et al. (20) do not include nudges. However, according to Newall (18), researchers in the field of gambling should pay close attention to electronic gambling machines because they constitute a large part of “gambling’s public health costs.” He also highlights the importance of informing people who gamble about the real odds of winning. For their part, and in concordance with the subject of their article, Pennay et al. (20) suggest that researchers should study various populations and sports bar environments to guide forthcoming public health interventions.

The other four selected articles suggest future uses of nudges in gambling. Gainsbury et al. (11, 15) highlight the role of researchers who study nudges in gambling. The 2018 article mentions that researchers should complete more studies to evaluate nudges in gambling and the effectiveness of interventions on different groups (11). The 2020 article suggests that researchers identify and investigate persuasive designs in gambling and nudges (15). Also, Murch and Clark (17) consider that nudges can establish a bridge between research findings and gambling policies. Gainsbury et al. (15) recommend that operators in the gambling industry detect people who gamble at “moderate-risk” and then send them personalized dynamic messages “about consumer protection tools.” According to Newall and Rockloff (19), operators could also redesign the deposit limit tool “by removing the dropdown list’s suggested limits and by getting gamblers to input their own limits.”

## 4 Discussion

To our knowledge, this is the first scoping review whose aim that synthesizes scientific peer-reviewed studies focusing directly on both nudge theory and gambling. Conducting this scoping review revealed that the relationship between nudge theory and gambling is frequently related to the presence of dark nudges in gambling. These are harmful to people who gamble—particularly those who have a gambling disorder—as they obstruct their decision-making process. In contrast, the development and implementation of beneficial nudges could reduce gambling harms and promote responsible gambling. While nudges are relatively easy to implement due to their low cost and subtlety, the fact that they often involve many gambling stakeholders can complicate the task.

Two authors in our study selection each have two articles in which they are the first author (11, 15, 18, 19), thereby limiting our exposure to diverse views about nudges in gambling. As for article type, our selection is composed of only one original research study, which mainly concerns dark nudges (20). For that matter, two of the six articles are mostly about dark nudges (18, 20), while four of the six articles are from Australia (11, 15, 19, 20).

These limitations related to diversity in our study selection indicate a deficiency of studies (especially regarding peer-reviewed original research) on nudges and gambling. Future studies should test nudges on real people who gamble, including experts by experience and/or people with a gambling disorder (22), using realistic machines or platforms to ensure ecological validity (17). Maximizing the positive effects of this process would necessitate involving different stakeholders (11, 15, 18).

In addition, it is our opinion that ensuring a mutual understanding of the different concepts related to nudge theory requires that the scientific vocabulary be more uniform. Indeed, while developing our scoping review search strategy we observed that some articles use the terms “nudges” or “nudging” to discuss factors that harm the decision-making process, while these factors are in fact dark nudges. Examples include the articles of Carter and Hall (23) and Deans et al. (24), which use the term “nudge” when discussing features that aim to influence people to continue gambling. To avoid confusion, we believe that the terms “dark nudge” or “sludge” could be used instead in this context. We also observed that while some articles mention gambling features that could be considered nudges or dark nudges, none mention nudge theory or the concept of nudge itself. An example is the article of Graydon et al. (16), whose research about “losses disguised as wins” never mentions dark nudges. It is the commentary of Murch and Clark (17), included in our study selection, that links the two concepts. We also found original research articles that we consider to be related to the definition of nudges, but which do not mention this theoretical link. They address the concepts of dynamic warning messages (25), volatility warnings (26), and behavioral feedback (27) in gambling. However, it is important to highlight that nudge theory is a recent development, which may explain the lack of terminology, uniformity, and knowledge of these concepts within the scientific community. In conclusion, our review emphasizes the connection between nudge theory and gambling, emphasizing the detrimental effects of dark nudges. While beneficial nudges can mitigate gambling’s harms and contribute to harm minimization, the involvement of myriad stakeholders adds complexity to their implementation. The study’s limitations underscore the need for diverse and peer-reviewed research. A standardized vocabulary is crucial for clarity in discussions and research about nudges and gambling.

### 4.1 Strengths and limitations

This scoping review draws a systematic portrait of, and synthetizes the literature regarding, nudges and gambling. It also distinguishes terminology within nudge theory (i.e., nudge, sludge, dark nudge) and highlights the need for uniformity in the vocabulary of this emerging field. However, as this scoping review included only published articles in peer-reviewed journals, gray literature was not included. Furthermore, only articles written in English were included, which may have led to an underestimation of the number of articles linking nudges and gambling.

## 5 Conclusion

This scoping review asserts the promising utility of nudges to lead people who gamble toward safer and more responsible gambling practices. It also addresses the harmful side of nudges, dark nudges, which are used in gambling to exploit gamblers. Future research should empirically examine the effectiveness of nudges to gain a deeper understanding of their impact on individuals who engage in gambling. Such insights are essential for the crafting of well-informed policies that efficiently promote responsible gambling. Future research should also focus on the impacts of dark nudges, and test whether reducing dark nudges (or adding nudges) fosters safer gambling. With this objective in mind, it is imperative that all stakeholders implicated in gambling contribute to this process.

## Data availability statement

The raw data supporting the conclusions of this article will be made available by the authors, without undue reservation.

## Author contributions

M-ÈF: Writing – original draft, Writing – review & editing, Conceptualization, Data curation, Formal analysis, Investigation, Methodology. SA-C: Supervision, Validation, Writing – review & editing. A-MA: Writing – review & editing. MB: Conceptualization, Funding acquisition, Project administration, Supervision, Validation, Writing – review & editing.
